# Exploring potential biomarkers and therapeutic targets of long COVID-associated inflammatory cardiomyopathy

**DOI:** 10.3389/fmed.2023.1191354

**Published:** 2023-06-29

**Authors:** Peng Qi, Mengjie Huang, Haiyan Zhu

**Affiliations:** ^1^Department of Emergency, First Medical Center of Chinese PLA General Hospital, Beijing, China; ^2^Department of Nephrology, First Medical Center of Chinese PLA General Hospital, Beijing, China

**Keywords:** COVID-19, long COVID, inflammatory cardiomyopathy, bioinformatic analysis, differentially expressed genes

## Abstract

**Background:**

The negative impact of long COVID on social life and human health is increasingly prominent, and the elevated risk of cardiovascular disease in patients recovering from COVID-19 has also been fully confirmed. However, the pathogenesis of long COVID-related inflammatory cardiomyopathy is still unclear. Here, we explore potential biomarkers and therapeutic targets of long COVID-associated inflammatory cardiomyopathy.

**Methods:**

Datasets that met the study requirements were identified in Gene Expression Omnibus (GEO), and differentially expressed genes (DEGs) were obtained by the algorithm. Then, functional enrichment analysis was performed to explore the basic molecular mechanisms and biological processes associated with DEGs. A protein–protein interaction (PPI) network was constructed and analyzed to identify hub genes among the common DEGs. Finally, a third dataset was introduced for validation.

**Results:**

Ultimately, 3,098 upregulated DEGs and 1965 downregulated DEGs were extracted from the inflammatory cardiomyopathy dataset. A total of 89 upregulated DEGs and 217 downregulated DEGs were extracted from the dataset of convalescent COVID patients. Enrichment analysis and construction of the PPI network confirmed VEGFA, FOXO1, CXCR4, and SMAD4 as upregulated hub genes and KRAS and TXN as downregulated hub genes. The separate dataset of patients with COVID-19 infection used for verification led to speculation that long COVID-associated inflammatory cardiomyopathy is mainly attributable to the immune-mediated response and inflammation rather than to direct infection of cells by the virus.

**Conclusion:**

Screening of potential biomarkers and therapeutic targets sheds new light on the pathogenesis of long COVID-associated inflammatory cardiomyopathy as well as potential therapeutic approaches. Further clinical studies are needed to explore these possibilities in light of the increasingly severe negative impacts of long COVID.

## Background

**Figure 1:**
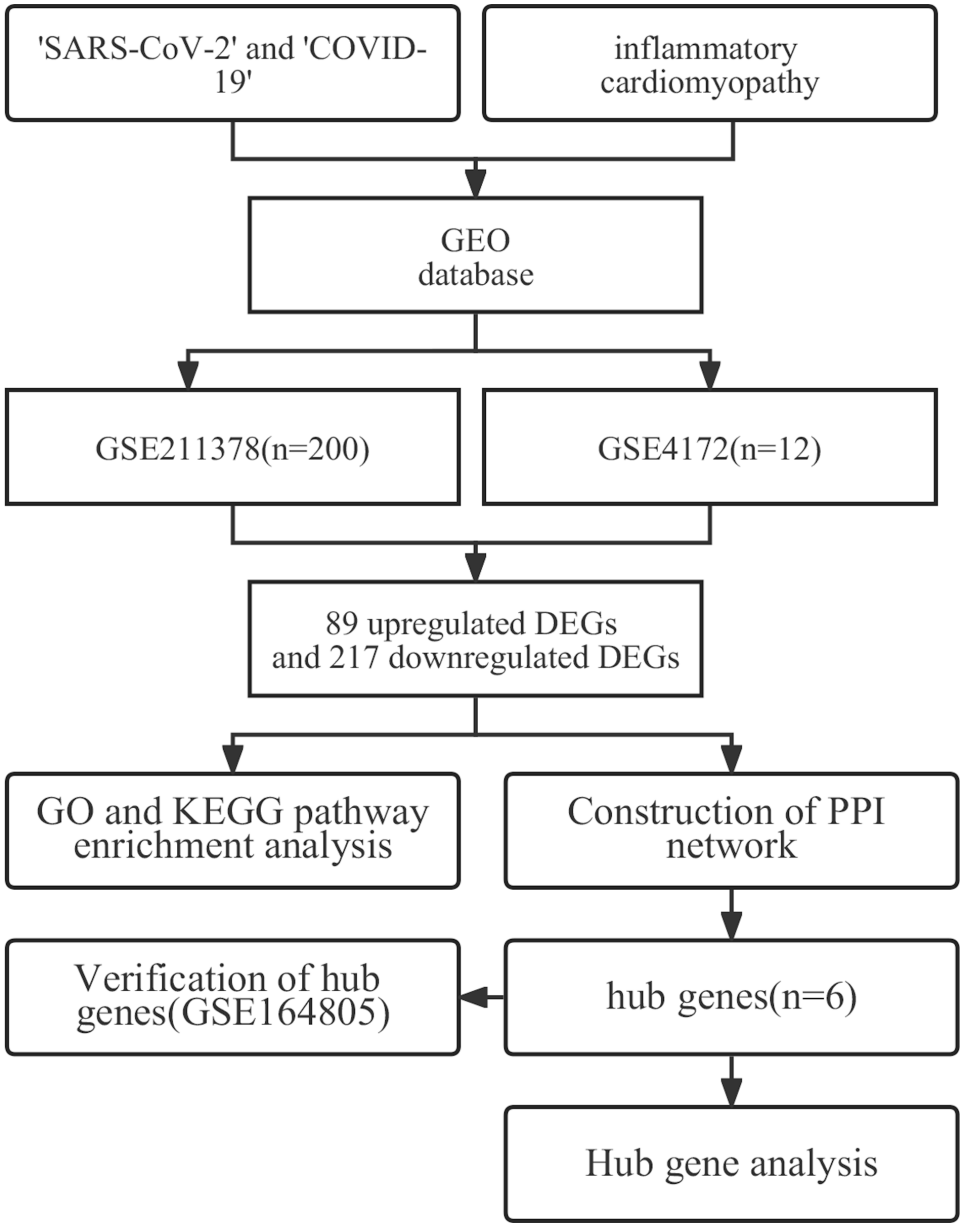
The workflow used in this study to explore potential biomarkers and therapeutic targets of long COVID-associated inflammatory cardiomyopathy.

COVID-19 is the disease caused by severe acute respiratory syndrome coronavirus 2 (SARS-CoV-2); this disease has a significant impact on global public health and the health of individuals (1, 2). As of February 2023, the World Health Organization reported 750 million confirmed cases of COVID-19, including more than 6.8 million fatal cases. According to the World Health Organization, 80–85% of COVID-19 infections are mild or asymptomatic, with recovery similar to that of seasonal influenza. However, an increasing number of reports indicate that many patients experience persistent negative effects after recovery from the initial stage of the disease, resulting in long-term health problems (3, 4). This phenomenon was first described on Twitter as ‘long COVID’ in May 2020; other reports have described it as ‘postacute sequelae of COVID-19’ (5) or ‘chronic COVID syndrome’ (6). Long COVID is a multisystem disease that occurs in at least 10% of COVID-19 infections. More than 200 symptoms affecting multiple organ systems have been identified (5). Fatigue, dyspnea, cough, loss of sense of smell, brain fog and dysgeusia are the most common symptoms of long COVID, but there have also been reports of organ-specific injuries involving the cardiovascular and neuropsychiatric systems, as well as diabetes symptoms (7–10). Recent studies have advanced many hypotheses regarding the pathogenesis of long COVID (11–18), but the exact etiology of long COVID is still unclear. The importance of developing and verifying biomarkers that can be used to diagnose long COVID cannot be overemphasized (5). Such biomarkers would not only aid in diagnosis but also potentially provide targets for treatment and prevention. Inflammatory cardiomyopathy is one of the more common components of long COVID (19, 20). Typical symptoms and signs of inflammatory cardiomyopathy include chest pain, dyspnea, fatigue, palpitations, syncope, and cardiogenic shock. It can also manifest as sudden cardiac death (21), accounting for approximately 10% of sudden cardiac deaths in young individuals (age < 35 years) (22). Inflammatory cardiomyopathy is mainly mediated by viral infection but can also be induced by systemic immune-mediated disease (23). In a prospective study, 60% of convalescent COVID-19 patients had myocardial inflammation, and 78% had abnormal cardiac magnetic resonance imaging results (24). In another study involving 153,760 patients with COVID-19, the patients exhibited an increased cardiovascular disease event burden for up to 12 months, even those patients without any cardiovascular disease basis (10). These data suggest that a deeper understanding of the pathogenesis of cardiovascular disease in convalescent COVID-19 patients is needed, and the negative challenges posed by long COVID urgently require treatment approaches that are still lacking (25, 26). To explore the potential biomarkers and therapeutic targets of long COVID-related inflammatory cardiomyopathy, we screened Gene Expression Omnibus to find appropriate datasets and screened out DEGs. To explore the basic molecular mechanism of the biological processes of the DEGs, functional enrichment analysis was performed. Then, a protein–protein interaction network was constructed to obtain hub genes. Finally, a third dataset was used to verify and make reasonable inferences. The workflow of this study is shown in Figure 1. We used bioinformatics techniques to mine more valuable information from the raw data in public databases to inform the subsequent experimental research design. This approach can also provide a better understanding of the mechanism of long COVID-related inflammatory cardiomyopathy and more effectively promote rapid diagnosis and drug treatment in the future.

## Methods

### Microarray data

We searched the GEO database with ‘SARS-CoV-2’ and ‘COVID-19’ as keywords, with ‘Experiment type’ limited to ‘Expression profiling by array’, and ‘Organism’ limited to ‘*Homo sapiens*’. From the original literature, we selected datasets that included both convalescent COVID-19 patients and healthy controls. Then, the datasets that met the principles of randomized controls and ethical requirements were retained, and finally, the GSE211378 dataset was selected. This dataset used the GPL32571 nCounter Host Response Panel v1.0, which included 160 convalescent COVID-19 patients (age:49.3 ± 6.2) with molecular or serological laboratory evidence of COVID-19 in the past and who had fully recovered from COVID-19. Except for loss of taste or smell, there is no other COVID-19 symptom that lasts for ≥28 days, or molecular detection that was negative for ≥14 days after recovery, and 40 healthy controls (age:50.9 ± 14.5) were selected. Then, the same search strategy was used to search for ‘inflammatory cardiomyopathy’ as the keyword, and the ‘GSE4172’ dataset, which included 8 patients with inflammatory cardiomyopathy. Inflammatory cardiomyopathy was confirmed through endocardial myocardial biopsy based on clinical, morphological and functional diagnostic criteria. The functional classification of the cases according to New York Heart Association was II. Four healthy controls were selected. The platform used was GPL570 [HG-U133_Plus_2] Affymetrix Human Genome U133 Plus 2.0 Array. The two datasets were downloaded through R software (version 4.2.1),^1^ and the original data from the two datasets were read using the ‘affy’ package (27). The RMA algorithm was used for background correction and data normalization.

### Identification of DEGs

The ‘limma’ package (28) in R was used to group and label the disease groups and the healthy control groups in the two datasets. The DEGs between the groups were obtained. Assuming that the differences between convalescent COVID-19 patients and normal controls might be relatively small, the criteria were set as (1) | log2 (fold-change)| > 0.1 and (2) *p* < 0.05 to screen out more meaningful differentially expressed genes. The genes that met these conditions were identified as having significant differences (28, 29). Among the selected DEGs, genes with logFC >0.1 were considered to be upregulated, while genes with logFC < −0.1 were considered to be downregulated.

### Go and KEGG pathway enrichment analysis of DEGs

GO functional analysis is a systematic method for gene annotation in the context of RNA and protein expression and is a powerful bioinformatics tool for classifying gene expression and its characteristics (30). KEGG is an online database of cellular, enzymatic and biochemical pathways that is used to determine which pathways may be impacted by changes in gene expression (31). The clusterProfiler package (32) in R was used for DEG enrichment analysis. The ‘org.Hs.eg.db’ package in R was used for ID conversion (33), and the ‘ggplot2’ package was used to visualize the enrichment analysis results.

### Construction of the PPI network

The DEGs common to the two datasets were obtained by Venn diagram analysis, and then the co-upregulated DEGs and co-downregulated DEGs were uploaded to the STRING database (34)^2^ to construct the PPI network, which is helpful for mining the core regulatory genes. Finally, the STRING results were imported into Cytoscape software (35) to test the potential correlation between these DEGs, and the results were outputted as a visual molecular interaction network.

### Screening of hub genes

Hub genes are defined as those proteins or DEGs that have higher degrees of association with others. The cytoHubba plug-in (36) in Cytoscape was used to identify hub genes among the common DEGs. Different plug-in algorithms may have different selection bias. Therefore, the DMNC, MCC, EPC, and MNC algorithms were used to calculate the hub genes, and the genes identified by all of the algorithms were defined as the final hub genes. The results are visualized in a Venn diagram.

### Analysis of hub genes

The Human Protein Atlas (37)^3^ is a public database of gene expression profiles in different human organs from which the basic RNA and protein expression levels of specific genes can be identified. The PANTHER classification system (38)^4^ has a comprehensive, annotated gene family system library that can classify proteins (and their genes). The selected hub genes were analyzed using the above databases.

### Verification of hub genes

Some reports have noted that the persistence of the virus may be a driving factor for long COVID, and it has even been shown that active virus or viral components can still be found in convalescent COVID-19 patients 12 months after the initial infection. In addition, inflammatory cardiomyopathy is mainly mediated by viral infection. Regardless of whether long COVID-associated inflammatory cardiomyopathy is mediated only by COVID-19 infection or also by the immune-mediated response and inflammation, the exact mechanism is still unclear. To further clarify this mechanism, we introduce data from a third set of COVID-19-infected patients for verification.

## Results

### Identification of DEGs

**Figure 2:**
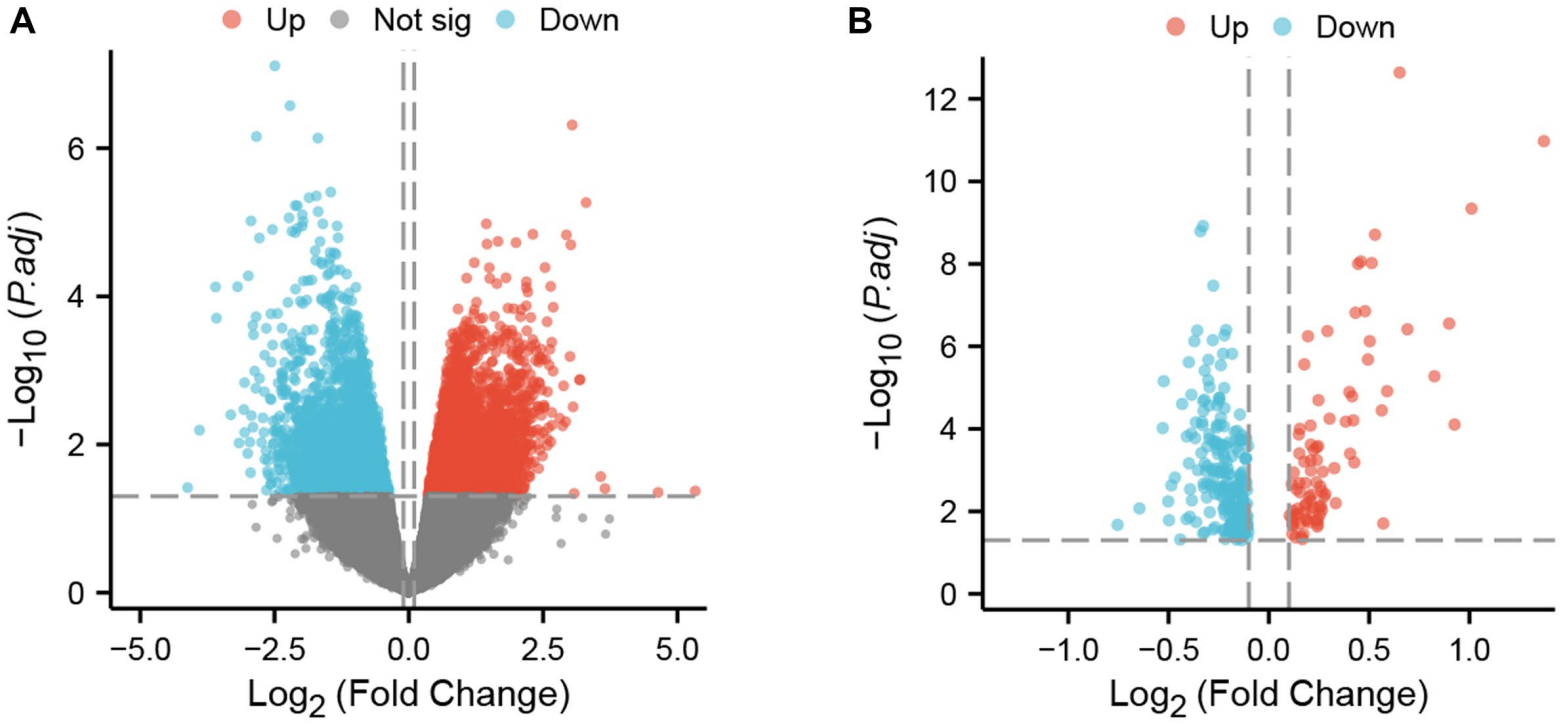
DEGs in two datasets. **(A)** Volcano plot of GSE4172; red represents upregulated genes, blue represents downregulated genes, and gray represents nonsignificantly expressed genes. **(B)** Volcano plot of GSE211378. The criteria for statistically significant differences for DEGs were adjusted |log2(FC)| > 0.1 and *p* < 0.05 for expression.

The two datasets were processed by the ‘limma’ package in R. In the GSE4172 dataset of inflammatory cardiomyopathy, the number of genes conforming to (1) log2 (fold-change) > 0.1 and (2) *p* <0.05 was 3,098, which was defined as upregulated DEGs. The number of genes conforming to (1) log2 (fold-change) < −0.1 and (2) *p* < 0.05 was 1965; these genes were defined as downregulated DEGs. Among the convalescent COVID-19 patients in the GSE211378 dataset, the number of genes conforming to (1) log2 (fold-change) > 0.1 and (2) *p* < 0.05 was 89; these were defined as upregulated DEGs. The number of genes conforming to (1) log2 (fold-change) < −0.1 and (2) *p* < 0.05 was 217; these genes were defined as downregulated DEGs. The results of differential analysis were visualized by the ‘ggplot2’ package (Figure 2).

### Go and KEGG pathway enrichment analyses of DEGs

**Figure 3:**
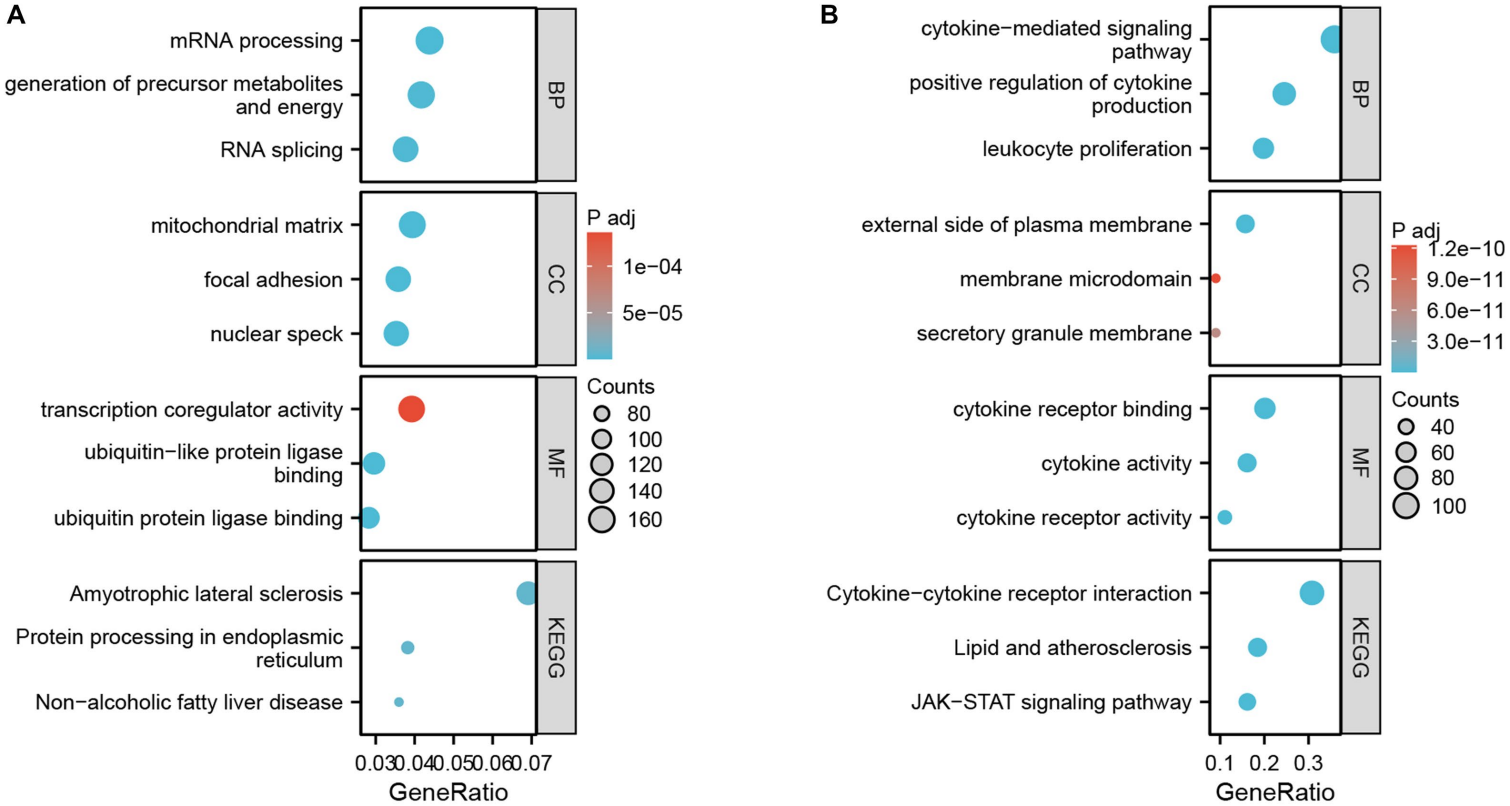
GO terms and KEGG pathway enrichment. **(A)** Details of GO terms and KEGG pathway enrichment for GSE4172. The colored dots represent the *p* value for that term, with red representing greater significance. The size of the dot represents the number of genes involved. **(B)** Details of GO terms and KEGG pathway enrichment for GSE211378.

Enrichment analysis of DEGs between the two datasets was performed with the clusterProfiler package in R. The biological process (BP) terms enriched among the DEGs in inflammatory cardiomyopathy (GSE4172) were mainly mRNA processing, generation of precursor metabolites and energy, RNA splicing, proteasome-mediated ubiquitin-dependent protein catabolic process and regulation of mRNA metabolic process. The enriched cell component (CC) terms were mainly mitochondrial matrix, nuclear speck, focal adhesion, cell-substrate junction and spliceosomal complex. The enriched molecular function (MF) terms were mainly ubiquitin protein ligase binding, ubiquitin-like protein ligase binding, transcription coregulator activity, oxidoreduction-driven active transmembrane, transporter activity and cytochrome-c oxidase activity. The enriched KEGG pathways were mainly nonalcoholic fatty liver disease, amyotrophic lateral sclerosis, protein processing in endoplasmic reticulum, Alzheimer’s disease and Huntington’s disease. The BP terms enriched among the DEGs of convalescent COVID-19 patients in GSE211378 were mainly cytokine-mediated signaling pathway, positive regulation of cytokine production, leukocyte proliferation, regulation of leukocyte proliferation and positive regulation of leukocyte proliferation. The enriched CC terms were mainly external side of plasma membrane, secretory granule membrane, membrane microdomain, membrane raft and plasma membrane signaling receptor complex. The enriched MF terms were mainly cytokine receptor binding, cytokine activity, cytokine receptor activity, immune receptor activity and receptor ligand activity. The enriched KEGG pathways were mainly cytokine–cytokine receptor interaction, lipid and atherosclerosis, JAK–STAT signaling pathway, viral protein interaction with cytokine and cytokine receptor and Th17 cell differentiation. Thus, many of the DEGs of the convalescent COVID-19 patients are related to cytokines, and research on the mechanism of long COVID can focus on this aspect. The enrichment results for the two datasets are visualized in the form of bubbles (Figure 3) and listed in a three-line table format (Tables 1, 2).

**Table 1:** Details of GO terms and KEGG pathway enrichment in GSE4172.

Ontology	ID	Description	GeneRatio	BgRatio	*p* value	*p*. Adjust
BP	GO:0006397	mRNA processing	164/3742	500/18800	4.08e-12	2.57e-08
BP	GO:0006091	Generation of precursor metabolites and energy	156/3742	494/18800	3.31e-10	1.04e-06
BP	GO:0008380	RNA splicing	141/3742	438/18800	5.6e-10	1.17e-06
BP	GO:0043161	Proteasome-mediated ubiquitin-dependent protein catabolic process	133/3742	414/18800	1.99e-09	3.14e-06
BP	GO:1903311	Regulation of mRNA metabolic process	101/3742	294/18800	3.65e-09	4.6e-06
CC	GO:0005759	Mitochondrial matrix	153/3883	473/19594	5.08e-11	1.4e-08
CC	GO:0016607	Nuclear speck	137/3883	411/19594	5.2e-11	1.4e-08
CC	GO:0005925	Focal adhesion	139/3883	419/19594	5.5e-11	1.4e-08
CC	GO:0030055	Cell-substrate junction	140/3883	428/19594	1.42e-10	2.71e-08
CC	GO:0005681	Spliceosomal complex	76/3883	194/19594	3.46e-10	5.28e-08
MF	GO:0031625	Ubiquitin protein ligase binding	108/3824	298/18410	4.02e-10	2.98e-07
MF	GO:0044389	Ubiquitin-like protein ligase binding	113/3824	317/18410	4.97e-10	2.98e-07
MF	GO:0003712	Transcription coregulator activity	150/3824	497/18410	3.4e-07	0.0001
MF	GO:0015453	Oxidoreduction-driven active transmembrane transporter activity	32/3824	72/18410	5.13e-06	0.0015
MF	GO:0004129	Cytochrome-c oxidase activity	13/3824	19/18410	9.97e-06	0.0022
KEGG	hsa04932	Non-alcoholic fatty liver disease	64/1779	155/8164	2.8e-08	6.86e-06
KEGG	hsa05014	Amyotrophic lateral sclerosis	123/1779	364/8164	4.58e-08	6.86e-06
KEGG	hsa04141	Protein processing in endoplasmic reticulum	68/1779	171/8164	6.16e-08	6.86e-06
KEGG	hsa05010	Alzheimer disease	124/1779	384/8164	6.82e-07	4.19e-05
KEGG	hsa05016	Huntington disease	103/1779	306/8164	7.24e-07	4.19e-05

**Table 2:** Details of GO terms and KEGG pathway enrichment in GSE211378.

Ontology	ID	Description	GeneRatio	BgRatio	*p* value	*p*. Adjust
BP	GO:0019221	Cytokine-mediated signaling pathway	107/298	486/18800	1.85e-94	7.49e-91
BP	GO:0001819	Positive regulation of cytokine production	73/298	475/18800	3.89e-51	7.86e-48
BP	GO:0070661	Leukocyte proliferation	59/298	330/18800	5.31e-45	7.16e-42
BP	GO:0070663	Regulation of leukocyte proliferation	50/298	254/18800	3.08e-40	3.12e-37
BP	GO:0070665	Positive regulation of leukocyte proliferation	42/298	158/18800	1.04e-39	8.4e-37
CC	GO:0009897	External side of plasma membrane	47/299	455/19594	1.46e-25	4.82e-23
CC	GO:0030667	Secretory granule membrane	27/299	312/19594	3.65e-13	6.01e-11
CC	GO:0098857	Membrane microdomain	27/299	327/19594	1.12e-12	1.23e-10
CC	GO:0045121	Membrane raft	26/299	326/19594	6.56e-12	5.4e-10
CC	GO:0098802	Plasma membrane signaling receptor complex	25/299	313/19594	1.64e-11	1.08e-09
MF	GO:0005126	Cytokine receptor binding	60/298	272/18410	5.35e-51	2.8e-48
MF	GO:0005125	Cytokine activity	48/298	235/18410	5.11e-39	1.34e-36
MF	GO:0004896	Cytokine receptor activity	33/298	97/18410	4.62e-35	6.82e-33
MF	GO:0140375	Immune receptor activity	38/298	148/18410	5.22e-35	6.82e-33
MF	GO:0048018	Receptor ligand activity	50/298	489/18410	7.6e-26	7.95e-24
KEGG	hsa04060	Cytokine-cytokine receptor interaction	80/260	295/8164	9.7e-55	2.45e-52
KEGG	hsa05417	Lipid and atherosclerosis	48/260	215/8164	4.95e-28	6.26e-26
KEGG	hsa04630	JAK–STAT signaling pathway	42/260	162/8164	2.38e-27	2.01e-25
KEGG	hsa04061	Viral protein interaction with cytokine and cytokine receptor	34/260	100/8164	1.35e-26	8.55e-25
KEGG	hsa04659	Th17 cell differentiation	34/260	108/8164	2.53e-25	1.28e-23

### Construction of PPI network

**Figure 4:**
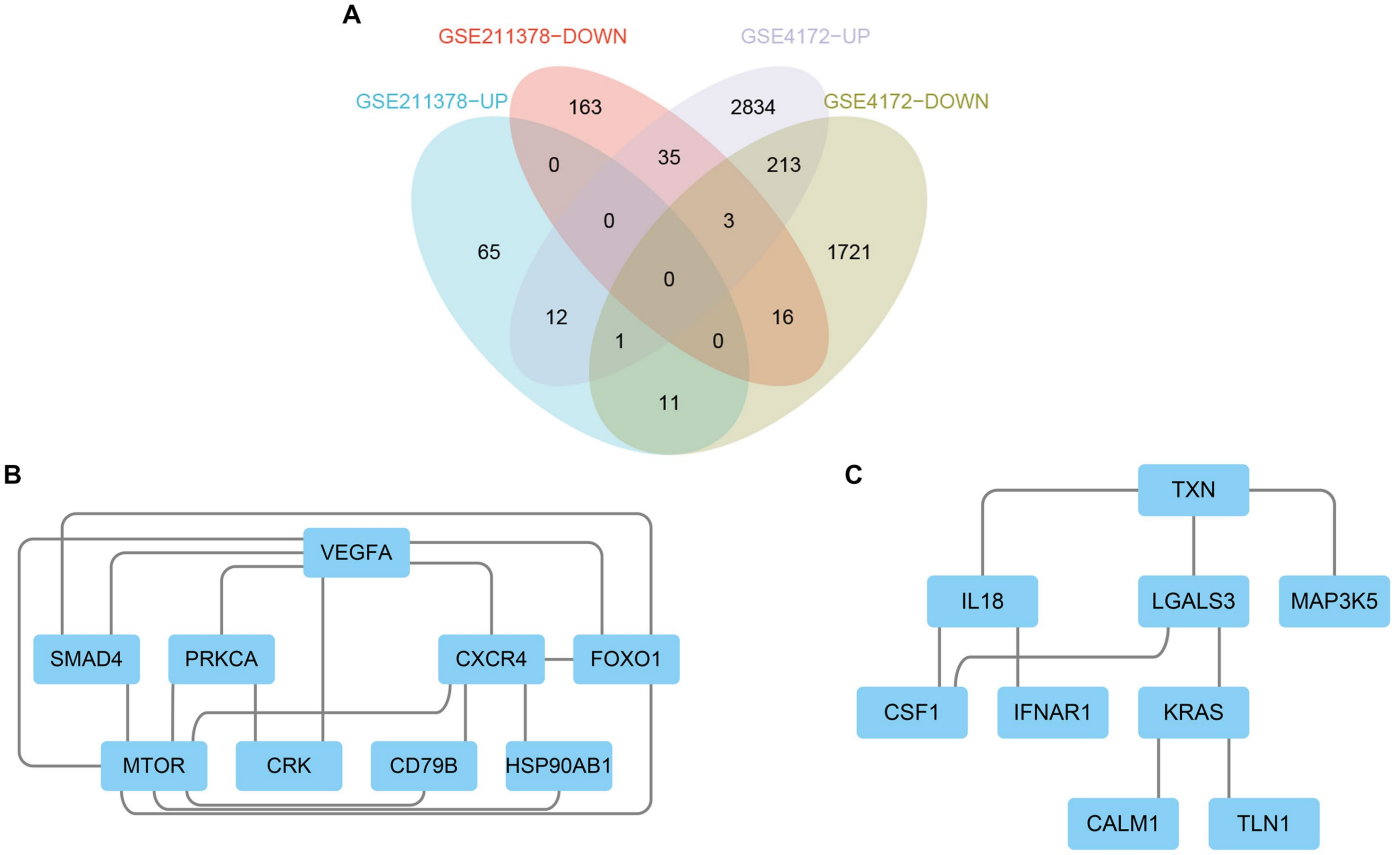
Venn diagram and PPI network. **(A)** The Venn diagram shows the co-DEGs of the two datasets. **(B)** A PPI network was constructed using co-upregulated genes. **(C)** A PPI network was constructed using co-downregulated genes.

The upregulated and downregulated DEGs between the two datasets were identified and then analyzed using the ‘VennDiagram’ package in R to obtain 12 upregulated common DEGs and 16 downregulated common DEGs (Figure 4A). Then, the obtained common DEGs were imported into the STRING database to generate a PPI network, and the generated information was imported into Cytoscape software to visualize the PPI network (Figures 4B,C).

### Screening of hub genes

**Figure 5:**
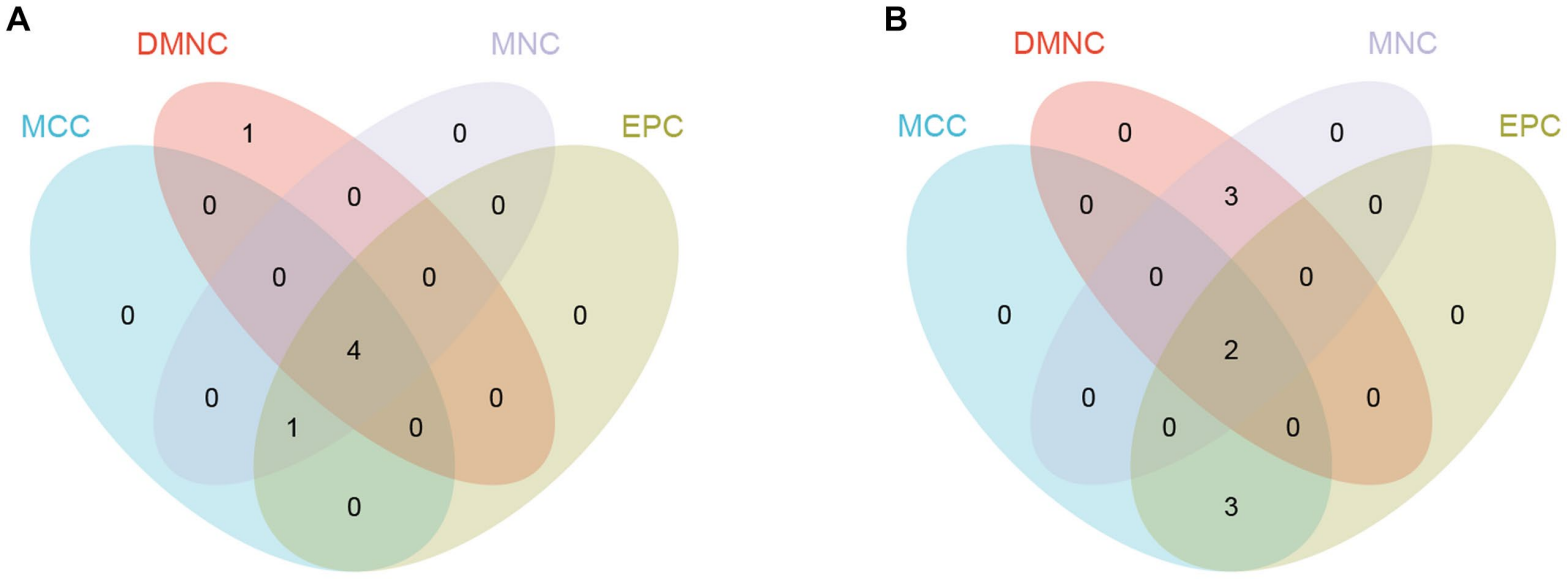
Hub genes were merged by the MCC, DMNC, MNC, and EPC algorithms. **(A)** VEGFA, FOXO1, CXCR4 and SMAD4 were upregulated hub genes. **(B)** KRAS and TXN were downregulated hub genes.

By using the cytoHubba plug-in of Cytoscape to extract hub genes, to avoid bias caused by different algorithms, four algorithms, DMNC, MCC, EPC, and MNC, were used to process the information, and the four groups of results were merged through the ‘VennDiagram’ package to confirm the final set of hub genes (Figure 5). VEGFA, FOXO1, CXCR4 and SMAD4 were identified as upregulated hub genes, and KRAS and TXN were identified as downregulated hub genes.

### Hub gene analysis

**Figure 6:**
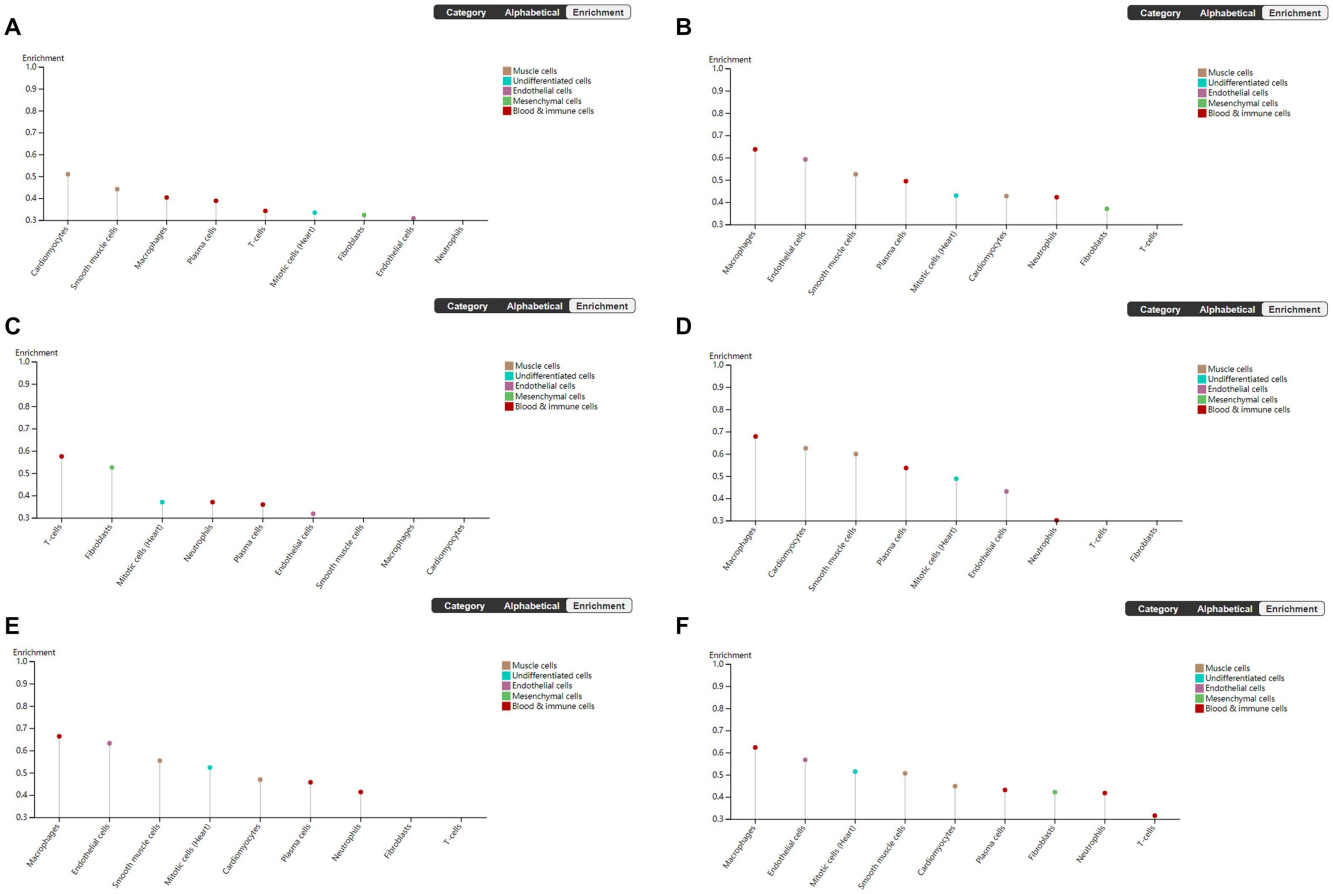
Human Protein Atlas was used to analyze hub genes. The plot shows the enrichment prediction score for each cell type profiled in heart muscle. This score is the mean correlation between the selected gene and the 3 reference transcripts selected to represent each cell type profiled within the tissue. **(A)** VEGFA, **(B)** FOXO1, **(C)** CXCR4, **(D)** SMAD4, **(E)** KRAS, and **(F)** TXN.

To explore the cell type specificity of hub genes in the human myocardium, the expression specificity of hub genes was predicted by using the Human Protein Atlas.^5^ The database prediction was generated by integrated network analysis of a large amount of publicly available RNAseq data; the results are shown in Figure 6. The PANTHER classification system^6^ can be used for one-stop annotation analysis of hub genes. The results are listed in Table 3.

**Table 3:** The PANTHER classification system of hub genes.

ID	Gene name	Gene ID	PANTHER family/subfamily	PANTHER protein class
VEGFA	Vascular endothelial growth factor A	HUMAN|HGNC = 12,680|UniProtKB=P15692	VASCULAR ENDOTHELIAL GROWTH FACTOR A (PTHR12025:SF5)	Growth factor
FOXO1	Forkhead box protein O1	HUMAN|HGNC = 3,819|UniProtKB = Q12778	FORKHEAD BOX PROTEIN O1 (PTHR45767:SF1)	Winged helix/forkhead transcription factor
CXCR4	C-X-C chemokine receptor type 4	HUMAN|HGNC = 2,561|UniProtKB=P61073	C-X-C CHEMOKINE RECEPTOR TYPE 4 (PTHR10489:SF594)	Cell adhesion molecule
SMAD4	Mothers against decapentaplegic homolog 4	HUMAN|HGNC = 6,770|UniProtKB = Q13485	MOTHERS AGAINST DECAPENTAPLEGIC HOMOLOG 4 (PTHR13703:SF63)	DNA-binding transcription factor
KRAS	GTPase KRas	HUMAN|HGNC = 6,407|UniProtKB=P01116	GTPASE KRAS (PTHR24070:SF388)	Small GTPase
TXN	Thioredoxin	HUMAN|HGNC = 12,435|UniProtKB=P10599	THIOREDOXIN (PTHR10438:SF18)	Oxidoreductase

### Verifying hub genes by ROC curve analysis

**Figure 7:**
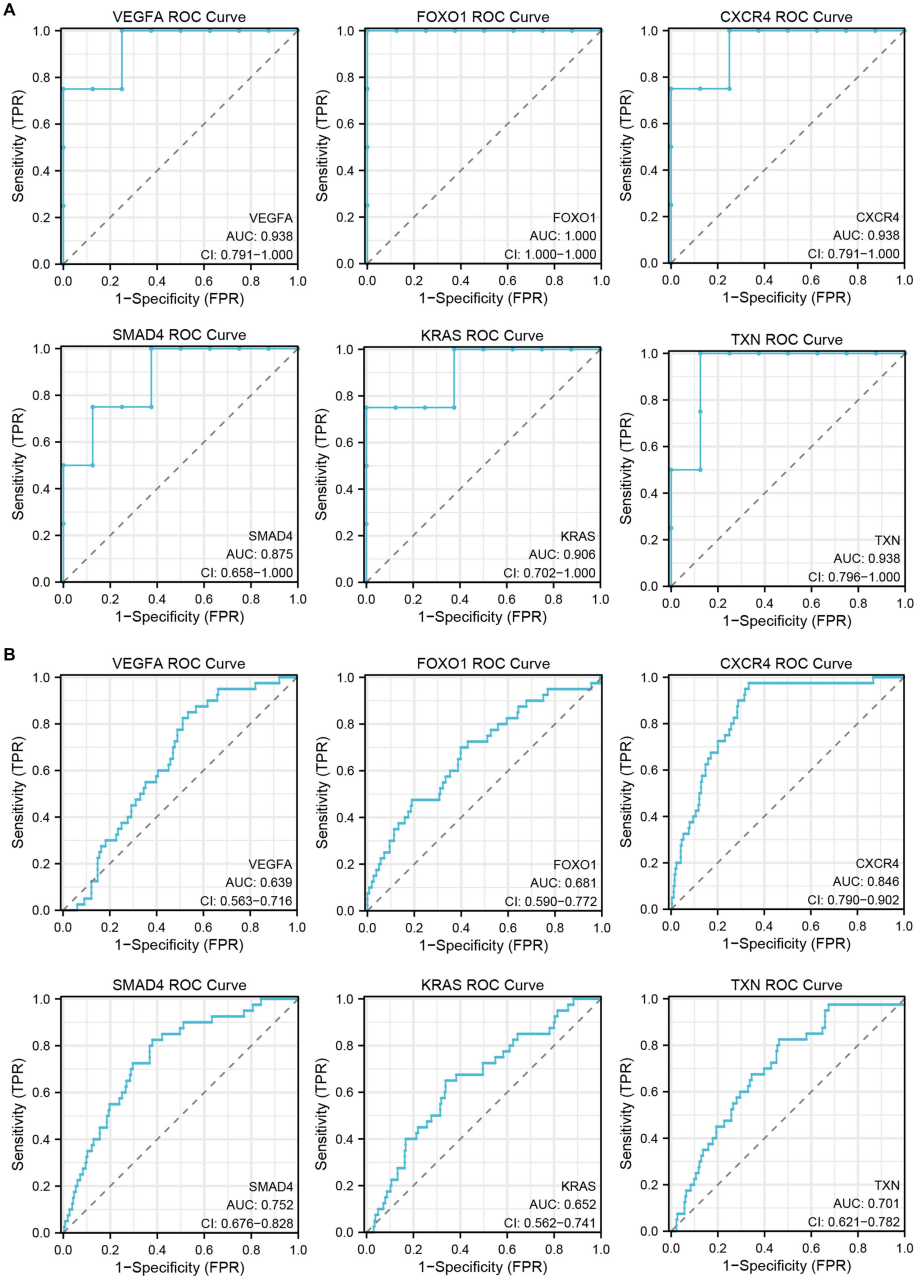
The GEO dataset was used to verify the diagnostic performance of hub genes. **(A)** ROC curve of hub genes in the GSE4172 dataset. **(B)** ROC curve of hub genes in the GSE211378 dataset.

To verify the diagnostic performance of the hub genes, we performed ROC curve analysis based on two GEO datasets. The results showed that all hub genes had good diagnostic performance in the inflammatory cardiomyopathy dataset, and the area under the ROC curve (AUC) was more than 85%. The relatively small difference between the patients who recovered from COVID-19 and the control group may be small may affect the diagnostic performance of the hub genes, but the results still showed that the area under the ROC curve of all hub genes in the COVID-19 recovery patient dataset was more than 60%. Therefore, we conclude that VEGFA, FOXO1, CXCR4, SMAD4, KRAS, and TXN are key genes in the common pathogenesis of long COVID and inflammatory cardiomyopathy, with great diagnostic value (Figure 7).

## Discussion

Long COVID may be caused by a variety of factors. A series of studies have proposed a variety of hypotheses about the pathogenesis of long COVID, including the persistence of viruses in tissues (12, 39), immune dysfunction (13, 14), destruction of the microbiota (40, 41), autoimmune response (15–18), coagulation and endothelial dysfunction (17, 18), and neurological signal dysfunction (42). With the increasing number of patients recovering from acute COVID-19 infection, the negative impact of long COVID on social life and individual health has become increasingly prominent. It has been reported that at 7 months after COVID-19 infection, patients’ quality of life remains diminished; 45.2% of convalescent COVID-19 patients need a reduced workload, and 22.3% of patients cannot return to work at all (7). This requires not only clinical trials on potential biomarkers and therapeutic targets for long COVID as soon as possible but also extensive public campaigns to make the public aware of the risks and consequences of long COVID (5). Although there are currently some diagnostic tools for long COVID-related diseases (43, 44), most of them are still being developed. There has been only some preliminary progress in the study of biomarkers of long COVID (45, 46). At present, there is an extreme lack of effective means to diagnose long COVID and evaluate its response to treatment. In this study, we performed bioinformatics analysis on long COVID-associated inflammatory cardiomyopathy. Through raw data mining and analysis, we obtained potential effective biomarkers and therapeutic targets. To verify and further explore the pathogenesis of long COVID-related inflammatory cardiomyopathy, we first introduced a third dataset for parallel verification. The GEO database was searched using ‘SARS-CoV-2’ and ‘COVID-19’ as keywords. From the original literature, we selected the datasets including both patients with COVID-19 infection and healthy controls, identified the datasets that met the principles of randomized control and ethical requirements, and finally selected the dataset GSE164805. The GSE164805 dataset includes 10 patients with COVID-19 infection and 5 healthy controls. By comparing the COVID-19 infection dataset (GSE164805) with the inflammatory cardiomyopathy dataset (GSE4172), we identified RRP12, FTSJ3, DCAF13, WDR46, DHX32, NLE1, POLR3B, NSUN4, HNRNPK, and HNRNPM as upregulated hub genes and RPS27, FAU, RPL35, RPL35A, RPL38, RPS11, RPS28, RPS19, RPL19, and RPL18 as downregulated hub genes. Through the comparison of hub genes between the three sets of datasets, it can be seen that the inflammatory cardiomyopathy-related targets are distinct between patients with COVID-19 infection and convalescent COVID-19 patients. Therefore, it is reasonable to speculate that long COVID-associated inflammatory cardiomyopathy is mainly attributable to the immune-mediated response and inflammation rather than direct viral infection of cells. Several other relevant reports support this theoretical inference (5, 47, 48). Second. we identified four upregulated hub genes, VEGFA, FOXO1, CXCR4, and SMAD4, and two downregulated hub genes, KRAS and TXN. Through Human Protein Atlas and the PANTHER classification system, we gained a preliminary and comprehensive understanding of the hub genes. We also conducted a detailed literature survey to further explore the hub genes. According to the literature, vascular endothelial growth factor A (VEGFA) is a homodimeric vasoactive glycoprotein and a key mediator of angiogenesis (49). Cardiomyocytes are the main source of VEGFA and the target of VEGFA (50–54). Studies have found that VEGFA plays a key role in triggering cardiac angiogenesis after acute myocardial infarction (55), but these studies are still limited to animal experiments. At present, there are no data on the expression and release of VEGFA in human cardiomyocytes. Further studies are needed to determine the mechanism of VEGFA in humans and whether this effect is valuable for patients recovering from COVID-19. Forkhead box O1 (FOXO1) is a representative member of the FOXO family and has key transcriptional regulatory activity (56). FOXO1 can regulate a variety of targets, such as genes involved in apoptosis and autophagy, antioxidant enzymes, cell cycle arrest genes, and metabolic and immunomodulators (57, 58). Due to its extensive endogenous expression, it may be an important target for the treatment and prevention of various diseases (59). C-X-C motif chemokine receptor 4 (CXCR4) is a cell chemokine receptor that plays an important role in development, hematopoiesis and immune surveillance through signal transduction induced by its ligand CXCL12. The pleiotropic effects of CXCR4 are well illustrated by the complexity of its biological functions and are mediated by mechanisms including receptor crosstalk, receptor and ligand isoforms, and unconventional ligands. Although the CXCL12–CXCR4 signaling pathway has been extensively studied, its atypical pathways remain to be fully elucidated (60). In addition, CXCR4 has become an interesting target in autoimmunity and inflammation based on its involvement in the chemotaxis of leukocytes to inflammatory sites (61, 62), the inappropriate retention (63) of activated innate inflammatory cells at inflammatory sites and its regulatory roles in autoimmune diseases (64, 65). Due to the great value of CXCR4 as a therapeutic target, the CXCR4 antagonist AMD3100 has also been approved by the US FDA (66, 67). Through ongoing research, CXCR4 has become a target for drug research and in the development of various diseases. SMAD family member 4 (SMAD4) is a member of the Smad family of signal transduction proteins, which are phosphorylated and activated by transmembrane serine–threonine receptor kinases through several pathways in response to transforming growth factor beta (TGF-β) signaling. At present, research on SMAD4 is mainly focused on its role as a target of tumor therapy (68), but some studies have confirmed that this protein also has activity in the late stage of COVID-19 infection. The mechanism of the association between long COVID and the development of cardiovascular disease may be through the activation of TGF-β signaling through the Smad pathway to induce subsequent myocardial fibrosis and scar formation (10). Since SMAD4 is abundantly expressed in the heart and vascular system during embryogenesis and plays a key role in normal cardiovascular development, the absence of SMAD4 leads to various cardiovascular developmental defects (69, 70). Thus, whether SMAD4 plays a specific role in inflammatory cardiomyopathy and cardiovascular disease during recovery from COVID-19 deserves further experimental research. The proto-oncogene KRAS is considered to be the most common gene driver of oncogenesis in human cancers (71). Overactivation of KRAS signaling has been shown to enhance the secretion of interleukin-6, which is necessary for tumorigenesis and tumor development (72). However, whether KRAS is involved in the development of inflammatory cardiomyopathy in patients recovering from COVID-19 and what role it plays in this process have not been published. Thioredoxin (TXN) is a hydrogen carrier protein that is widely expressed in organisms. TXN deficiency is associated with myocardial cell injury (73). This mechanism of this association may be the induction of autophagy in TXN-deficient cardiomyocytes, which leads to severe inflammation and myocardial cell damage (74). In summary, previous studies have, to a certain extent, confirmed the effectiveness and potential value of the hub genes identified in this study.

One limitation of our study is that the evidence for the identified potential biomarkers and therapeutic targets is limited to the theoretical level, and further experiments are needed to verify their activities. Another limitation is that there is no publicly available gene expression profile dataset of COVID-19-induced myocarditis. We did examine a dataset of parvovirus B19-related inflammatory cardiomyopathy. However, considering that the pathogenesis of inflammatory cardiomyopathy induced by COVID-19 infection and inflammatory cardiomyopathy in patients recovering from COVID-19 is not exactly the same, we used bioinformatics methods to analyze and extract the information hidden in the intergroup data, which will help to obtain effective potential biomarkers and therapeutic targets for long COVID-related inflammatory cardiomyopathy. Due to the particularity of SARS-CoV-2 infection, the development of many clinical studies has been limited to varying degrees. This makes the role of bioinformatics analysis more prominent in the study of COVID-19. Some recent studies (75) have also predicted potential therapeutic drugs for the ischemic cardiomyopathy associated with COVID-19 through bioinformatics methods. This research provides meaningful exploration methods for the study of long COVID-related diseases.

## Conclusion

Based on our bioinformatics analysis and previous research, this study identified potential targets (VEGFA, FOXO1, CXCR4, SMAD4, KRAS, TXN) that may be helpful for the diagnosis and treatment of long COVID-related inflammatory cardiomyopathy. The screening of these targets provides new directions for the study of the pathogenesis and treatment of long COVID-related inflammatory cardiomyopathy. Further clinical research is needed to explore these possibilities to address the increasingly serious negative impacts of long COVID.

## Data availability statement

The datasets presented in this study can be found in online repositories. The names of the repository/repositories and accession number(s) can be found in the article/supplementary material.

## Author contributions

PQ was the major contributor to the writing of the manuscript. MJH evaluated and revised the manuscript. HZ guided the writing of the manuscript and critically reviewed it. All authors contributed to the article and approved the submitted version.

## Funding

This research was supported by the National Key R&D Program of the Ministry of Science and Technology (2019YFC0119600), National Natural Science Foundation of China (82000631), Beijing Natural Science Foundation (7222169), Young Elite Scientist Sponsorship Program by CAST 2020QNRC001 (to MJH), and the Military Medical Youth Special Project of PLA General Hospital (QNF19035).

## Conflict of interest

The authors declare that the research was conducted in the absence of any commercial or financial relationships that could be construed as a potential conflict of interest.

## Publisher’s note

All claims expressed in this article are solely those of the authors and do not necessarily represent those of their affiliated organizations, or those of the publisher, the editors and the reviewers. Any product that may be evaluated in this article, or claim that may be made by its manufacturer, is not guaranteed or endorsed by the publisher.

## Footnotes

^1^ http://r-project.org/

^2^ http://string-db.org/

^3^ https://www.proteinatlas.org/

^4^ http://pantherdb.org/

^5^ https://www.proteinatlas.org/

^6^ http://pantherdb.org/

## Abbreviations

COVID: coronavirus disease
SARS-CoV-2: severe acute respiratory syndrome coronavirus 2
GEO: gene expression omnibus
PPI: Protein–protein interaction
DEG: differentially expressed gene
BP: biological process
CC: cell component
MF: molecular function
VEGFA: vascular endothelial growth factor A
FOXO1: forkhead box O1
CXCR4: C-X-C motif chemokine receptor 4
SMAD4: SMAD family member 4
KRAS: KRAS proto-oncogene
TXN: thioredoxin
